# Observations on Purging in Typhus

**Published:** 1812-08

**Authors:** 


*To the Editors of the Medical and Physical Journal.*

GENTLEMEN,

IN your Journal for June, I observed some observations on the utility of *extensive Purging in Typhus*, with two cases subjoined, to prove its beneficial effects, by Dr. Pigot. I think it a duty incumbent on every man, as far as lies in his power, to support truth, and expose falsehood; to encourage learning, and detect ignorance; particularly on subjects on which the health and lives of mankind depend. These alone are my motives for offering a few remarks on the cases mentioned. I should be very sorry that any young and unexperienced practitioner should be misled by the *seeming safety and efficacy* of this practice, and would still be more so, on seeing it attempted by any medical gentleman in *extensive practice;* as I am well convinced (if Typhus was as often to be met with as the cases recorded and called such by them) that it would make *greater havoc in the land than a French army.* In the first, Thomas Martin's case, I am quite surprised how it possibly should come under the denomination of Typhus. I read the case three or four times over, without perceiving a single symptom to confirm the least suspicion of Typhus, if so great an authority as Dr. Cullen's is to be attended to. The pulse is reported to be 104; this, in my opinion, by no means indicates Typhus; but, whether the pulse was full and hard, or weak and feeble, we are not informed; of course every one will will conclude that they were not feeble, else a person wishing to prove the utility of such a practice, would be very glad to lay hold of the slightest reed, to justify, in the smallest degree, his conclusion of this case being a Typhus, or rather to cover his own ignorance in making such an assertion. I see nothing else in the whole statement from which any conclusion might be drawn, except that the nurse found it necessary to use the straight waistcoat, which might have happened if the patient had been *intoxicated;* this indeed might be very improbable; still, from the statement of the case, and the duration of the illness, I should as soon suspect it as Typhus.

In the doctor's remarks on the case, we are told, that it amply shews how soon the *worst symptoms* of fever might be removed by the vigorous administration of purgatives. From what the doctor has given of the case to the public, I cannot, as I observed before, perceive one symptom of any sort to justify his calling it Typhus, even in its mildest state, much less in its worst.

From what I have observed in Typhus, when once formed, purgatives always and invariably raised the pulse; that they may reduce it, I am not able to say; but, from what I always observed, they had quite a contrary effect.

With respect to the other case (S. Hopley's), I have but the same observation to make with regard to the nature of the case; I can see nothing in it like Typhus. But leaving all arguments aside, with regard to the nature of the complaint, I refer to all the medical gentlemen in Great Britain for an impartial solution of the following question:

Can any person, whatever his complaint may be, take the *following purgatives* in the time prescribed?

| | Calomel. | Jalapii. | Magnes. Sulph. | | | Mannæ. | | | Tr. Jalap. |
|---|---|---|---|---|---|---|---|---|---|
| From | | | lb. | oz. | dr. | lb. | oz. | dr. | oz. |
| Jan. 7th to 8th | gr. x. | ʒſs. | 0 | 3 | 0 | 0 | 1 | 4 | |
| 9th | gr. x. | ʒſs. | 0 | 6 | 0 | 0 | 3 | 0 | |
| 10th | . . . . | . . . . | 0 | 6 | 0 | 0 | 3 | 0 | |
| 11th | . . . . | . . . . | 1 | 0 | 0 | 0 | 6 | 0 | 6 |
| 12th | . . . . | . . . . | 1 | 0 | 0 | 0 | 6 | 0 | 6 |
| 13th | . . . . | . . . . | 1 | 0 | 0 | 0 | 6 | 0 | 6 |
| 14th | . . . . | . . . . | 1 | 0 | 0 | 0 | 6 | 0 | 6 |
| | ℈j. | 1 dr. | 5 | 3 | 0 | 2 | 7 | 4 | 1 pint 8 oz. |

I should think that no man, acquainted with the nature of medicine and diseases, would hesitate a moment in giving an answer. I firmly believe that never one of Dr. Pigot's patients, patients, in the Chester Infirmary, had so much as *six pints* of the saline aperient mixture in the course of *four-and-twenty hours.* That any man took that quantity for four successive days, is, I think, a *matter of impossibility.* If you could find room for the above in some corner of your valuable Journal for the next month, you would greatly oblige,

Gentlemen,

Your most humble Servant,

*Chester, June* 13, 1812. MEDICUS.

